# *Agrobacterium rhizogenes*-Mediated Hairy Root Genetic Transformation Using *Agrobacterium* Gel Inoculation and *RUBY* Reporter Enables Efficient Gene Function Analysis in Sacha Inchi (*Plukenetia volubilis*)

**DOI:** 10.3390/ijms26062496

**Published:** 2025-03-11

**Authors:** Kai Lin, Li-Xin Lu, Bang-Zhen Pan, Xia Chai, Qian-Tang Fu, Xian-Chen Geng, Yi Mo, Yu-Chong Fei, Jia-Jing Xu, Meng Li, Jun Ni, Zeng-Fu Xu

**Affiliations:** 1 Guangxi Key Laboratory of Forest Ecology and Conservation, Guangxi Colleges and Universities Key Laboratory for Cultivation and Utilization of Subtropical Forest Plantation, College of Forestry, Guangxi University, Nanning 530004, China; linkai@st.gxu.edu.cn (K.L.); 2009392006@st.gxu.edu.cn (X.-C.G.);; 2 Key Laboratory of National Forestry and Grassland Administration on Cultivation of Fast-Growing Timber in Central South China, State Key Laboratory for Conservation and Utilization of Subtropical Agro-BioreSources, College of Forestry, Guangxi University, Nanning 530004, China; 3 CAS Key Laboratory of Tropical Plant Resources and Sustainable Use, Xishuangbanna Tropical Botanical Garden, Chinese Academy of Sciences, Mengla 666303, Chinafuqiantang@xtbg.ac.cn (Q.-T.F.)

**Keywords:** *Plukenetia volubilis*, hairy roots, *Agrobacterium* gel, *RUBY*, CRISPR-Cas9

## Abstract

*Plukenetia volubilis* L., a woody oilseed plant rich in α-linolenic acid, represents a promising source of polyunsaturated fatty acids. However, the lack of an efficient genetic transformation system has significantly hindered gene function research and molecular breeding in *P. volubilis*. In this study, we developed a highly efficient *Agrobacterium rhizogenes*-mediated hairy root transformation system for *P. volubilis* via the use of *Agrobacterium* gel in combination with the visually detectable *RUBY* reporter for gene function analysis in roots. The results indicate that the optimal transformation method involves infecting *P. volubilis* seedlings with *Agrobacterium* gel containing acetosyringone and inducing hairy root formation in perlite. This approach resulted in more than 18.97% of the seedlings producing positive hairy roots overexpressing the *RUBY* gene. Using this genetic transformation system, we successfully overexpressed the antimicrobial peptide-encoding gene *CEMA* in hairy roots, which enhanced the resistance of *P. volubilis* to *Fusarium oxysporum*. Furthermore, by combining this transformation system with the CRISPR-Cas9 tool, we validated the regulatory role of *PvoSHR* in the development of root epidermal cells in *P. volubilis*. Unexpectedly, a 123-bp DNA fragment from the T-DNA region of the *A. rhizogenes* Ri plasmid was found to be knocked in to the *P. volubilis* genome, replacing a 110-bp fragment of *PvoSHR* at CRISPR-Cas9 induced double-strand DNA breaks. Conclusively, this system provides a powerful tool for gene function research in *P. volubilis* and provides novel insights into the development of transformation and gene editing systems for other woody plants.

## 1. Introduction

*Plukenetia volubilis* L., commonly known as sacha inchi, is a perennial woody oilseed plant belonging to the Euphorbiaceae family and native to regions of South America, including Peru and Brazil [1]. The seeds of *P. volubilis* contain up to 40% oil, which is predominantly composed of polyunsaturated fatty acids, with ω-3, ω-6, and ω-9 fatty acids comprising more than 90% of the total composition. Notably, ω-3 fatty acids account for 45% to 52% of the oil content [2,3]. The oil of *P. volubilis* is known for various health benefits, including lowering blood lipids, reducing blood pressure, modulating immune function, decreasing the risk of cardiovascular diseases, and inhibiting *Staphylococcus aureus* on the skin. Consequently, it is widely utilized in industries such as food and cosmetics [1,4,5].

Essential candidate genes involved in the metabolism of α-linolenic acid [6,7], sex flower differentiation [8], and ricin synthesis metabolism [9] have been identified in *P. volubilis*. However, many critical functional genes cannot be further validated or utilized due to the lack of an efficient transformation system in *P. volubilis*. To overcome the regeneration bottleneck of stable genetic transformation, we employed *A. rhizogenes*-mediated genetic transformation to obtain transgenic hairy roots as an alternative approach to generate the regenerated transgenic plants of *P. volubilis*.

When *A. rhizogenes* infects plant explants, the T-DNA of the Ri plasmid is transferred and integrated into the host cell genome. This results in the expression of *rol* genes, which induces the formation of hairy roots in infected cells [10]. Transgenic hairy roots can be obtained more rapidly through *A. rhizogenes*-mediated transformation, typically within one month, than regenerated transgenic shoots through the transformation mediated by *A. tumefaciens* or *A. rhizogenes* [11]. Hairy roots provide a platform for target gene overexpression, silencing, and knockout, which enables the study of root gene function [12,13,14]. In some species, such as *Poncirus trifoliata*, *Malus domestica*, *Actinidia chinensis*, *Taraxacum kok-saghyz*, and *Dioscorea esculenta*, hairy roots can also regenerate shoots, ultimately leading to the production of complete transgenic plants [15,16,17]. *Agrobacterium*-mediated transformation can be categorized into two types: tissue culture-based transformation and *in planta* transformation, with the latter not requiring tissue culture [18]. The *in planta A. rhizogenes*-mediated transformation method eliminates the need for strict sterile culture conditions and labor-intensive tissue culture procedures, enabling the rapid production of transgenic hairy roots and chimeric plants.

This study innovatively combines *Agrobacterium* and hydroxypropyl methylcellulose (HPMC) to form an *Agrobacterium* gel for plant genetic transformation and develops an efficient and stable in planta *A. rhizogenes*-mediated hairy root transformation for *P. volubilis* via the visual reporter gene *RUBY*. Through the comparisons of exogenous additives, infection methods, and potting substrate types, we optimized the transformation system, which was subsequently applied to induce hairy roots in *P. volubilis* stem segments and other woody plants. Furthermore, this transformation system was used to investigate the functions of the insect antimicrobial peptide (cecropin A) and the melittin hybrid peptide-encoding gene *CEMA* (*cecropin A-melittin hybrid peptide*) [19,20] in relation to resistance to *F. oxysporum* infection, as well as *PvoSHR*, a homologous gene of the *SHORT ROOT* (*SHR*) in the radial cell differentiation of the root [21]. This genetic transformation system provides strong technical support for functional gene studies in *P. volubilis* and offers valuable insights for developing genetic transformation systems for hairy roots in other important tree species.

## 2. Results

### 2.1. Establishment of an in Planta A. rhizogenes Gel-Mediated Transformation System for P. volubilis

**Figure 1:**
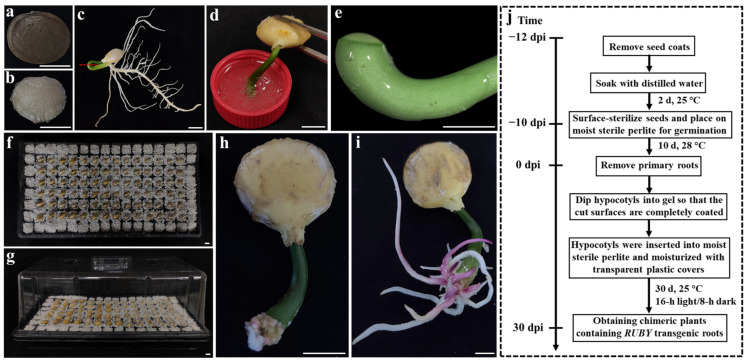
Establishment of an *A. rhizogenes*−mediated transformation system for *P. volubilis.* (**a**) *P. volubilis* seeds; (**b**) removal of the seed coats; (**c**) removal of the roots from the *P. volubilis* seedling (the red dashed line indicates the location for root removal); (**d**) dipping into *A. rhizogenes* gel; (**e**) *A. rhizogenes* gel covering the cut surface; (**f**) insertion into sterile wet perlite; (**g**) moisture retention using a transparent cover; (**h**) regeneration of roots and *RUBY* callus at the cut surface (*A. rhizogenes* co−cultivation for 10 days); (**i**) chimeric plants of *P. volubilis* containing *RUBY* transgenic roots (*A. rhizogenes* co−cultivation for 30 days); (**j**) schematic diagram of the technical process; bars = 1 cm.

In this study, we established a novel in planta *A. rhizogenes* gel-mediated transformation system for *P. volubilis* (Figure 1). The method involves infecting wound sites with *A. rhizogenes* gel and utilizing the visually detectable RUBY vector for the positive selection of hairy roots. The main steps were as follows: uncoated and surface-disinfected *P. volubilis* seeds were placed in a moist perlite to germinate for 10 days. After germination, the seedlings’ plume hooks were cut (Figure 1a–c), and the basal portions of the hypocotyl and primary root were discarded. The cut surface of the hypocotyls was then dipped into the *A. rhizogenes* gel (Figure 1d), ensuring the complete coverage of the wound (Figure 1e). The seedlings were subsequently inserted into perlite for moisture retention (Figure 1f,g) to induce hairy roots. After 10 days of *A. rhizogenes* co-cultivation, red callus tissue overexpressing *RUBY* began to form at the cut surface (Figure 1h). After 30 days of *A. rhizogenes* co-cultivation, chimeric *P. volubilis* plants containing *RUBY*-overexpressing hairy roots were obtained (Figure 1i). A flowchart of the transformation process is shown in Figure 1j.

The PCR results showed that the three genes of the *RUBY* expression cassette were detected in the transgenic hairy roots, whereas the streptomycin-resistance gene *SmR* [22] was not detected (Figure S1a). These results suggest that the *RUBY* gene has been stably integrated into the genome of the red hairy roots without contamination from *A. rhizogenes*. A qPCR analysis further revealed that the *RUBY* genes were expressed in red hairy roots, which led to high betalain accumulation (Figure S1b–d). In conclusion, these findings demonstrate the successful establishment of a stable in planta *A. rhizogenes* gel-mediated transformation system for *P. volubilis*.

### 2.2. Effects of Exogenous Additives, Infection Methods, and Potting Substrate Types on Transgenic Hairy Roots of P. volubilis

**Figure 2:**
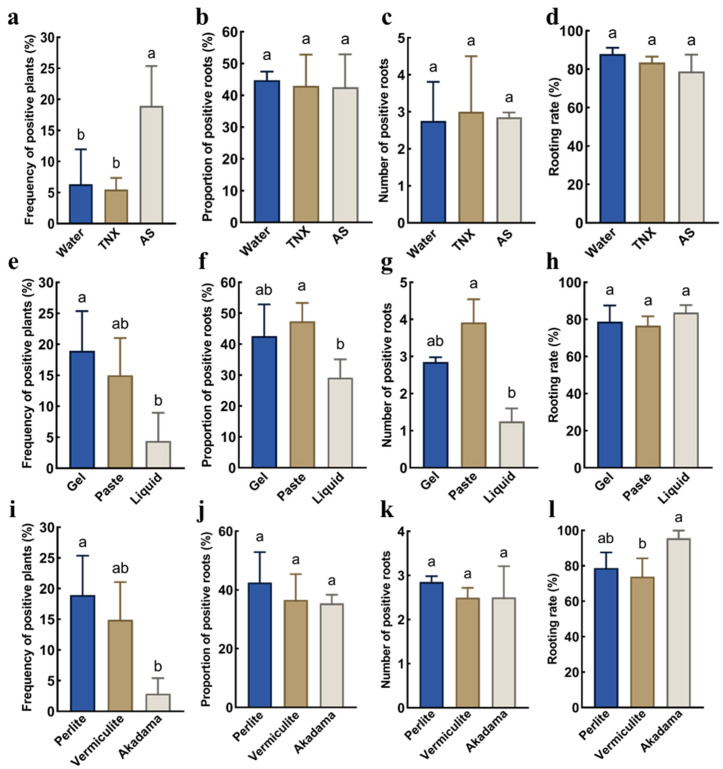
Effects of acetosyringone (AS), tenoxicam (TNX), inoculation method, and potting substrate type on transgenic hairy root formation in *P. volubilis.* (**a**–**d**) the effect of different exogenous additives on the frequency of positive plants, proportion of positive root, number of positive roots and rooting rate; (**e**–**h**) the effect of different inoculation methods on the frequency of positive plants, proportion of positive root, number of positive roots and rooting rate; (**i**–**l**) the effect of different potting substrate types on the frequency of positive plants, proportion of positive root, number of positive roots and rooting rate; different lowercase letters indicate significant differences at 0.05 level (*p* < 0.05).

To investigate the effects of different exogenous additives on the induction efficiency of transgenic hairy roots in *P. volubilis*, we first supplemented the *Agrobacterium* gel with acetosyringone (AS), tenoxicam (TNX), or water, respectively. AS is a natural phenolic compound secreted by injured plant cells, which enhances plant transformation efficiency by activating the expression of the *Agrobacterium Vir* genes [23]. TNX, an oxicam-type non-steroidal anti-inflammatory drug, has been shown to suppress plant immune responses and enhance *Agrobacterium* infection efficiency [24]. The results showed that the addition of AS increased the percentage of positive plants from 6.35% to 18.97%, whereas the percentage in the TNX treatment was only 5.48% (Figure 2a). No significant differences were observed among the three groups in terms of the proportion of positive roots, the number of positive roots, or rooting rate (Figure 2b–d). These findings suggested that AS significantly improved the efficiency of transgenic hairy root induction.

Next, we compared the transformation efficiency of the three infection methods: *Agrobacterium* gel, suspension, and paste (Figure S2). The results indicated that the percentage of positive plants, proportion of positive roots, and number of positive roots were significantly greater in the *Agrobacterium* gel and paste groups than in the *Agrobacterium* suspension group (Figure 2e–h). No significant differences were observed between the *Agrobacterium* gel and paste methods in terms of the percentage of positive plants, proportion of positive roots, number of positive roots, or rooting rate (Figure 2e–h). Given that *Agrobacterium* paste forms only after 1–2 days of culture post-plating, whereas a large amount of *Agrobacterium* gel can be prepared 10–20 min after adding HPMC to the *Agrobacterium* suspension, the *Agrobacterium* gel method is the preferred infection strategy.

Finally, we investigated the effects of different potting substrates (vermiculite, perlite and Akadamatsuchi) on the induction of hairy roots in *P. volubilis*. The results showed that the percentages of positive plants for vermiculite and perlite (14.93% and 18.97%, respectively) were significantly greater than that for Akadamatsuchi (2.90%), but Akadamatsuchi exhibited the highest rooting rate (95.51%) (Figure 2i–l). We postulated that Akadamatsuchi might promote the growth of adventitious roots at the hypocotyl cross-section of *P. volubilis*, but inhibit the regeneration of *RUBY* transgenic hairy roots. Furthermore, the percentage of positive plants and the number and proportion of positive roots were all greater in perlite than in vermiculite (Figure 2i–l). While perlite and vermiculite show no statistical difference in the percentage of positive plants or in the number and proportion of positive roots, vermiculite tends to adhere to hairy roots, making complete removal challenging. This adhesion can hinder the observation of disease phenotypes following pathogen inoculation. Additionally, vermiculite exhibits strong autofluorescence under red fluorescence excitation, which can disrupt the screening of hairy roots with fluorescent markers. Therefore, perlite is the optimal potting substrate for obtaining *P. volubilis* hairy roots.

### 2.3. Transformation of GUS and DsRed2 Reporter Genes via an in Planta Agrobacterium Gel Transformation System

**Figure 3:**
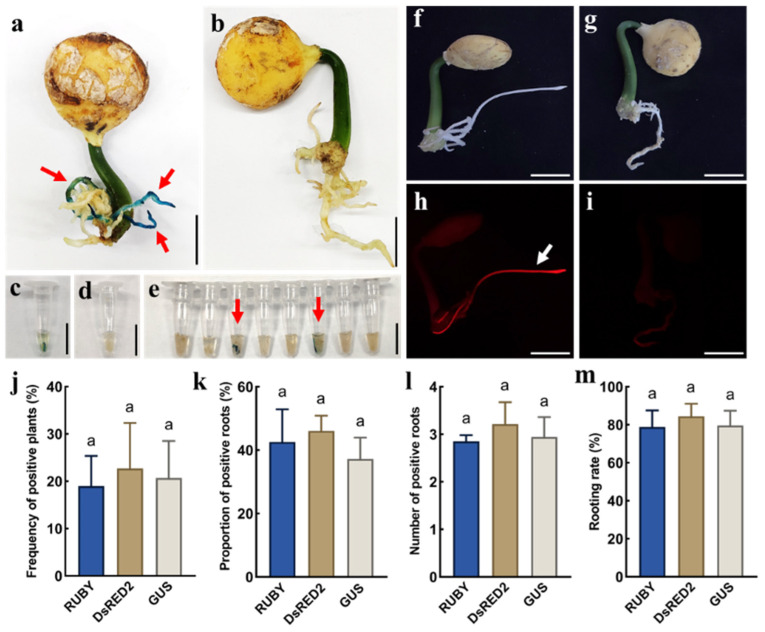
Transformation of *GUS* and *DsRed2* genes using the in planta *Agrobacterium* gel transformation system. (**a**) *P. volubilis* chimeric plant with *GUS*-transformed roots; (**b**) WT *P. volubilis* plant; (**c**) *GUS*-positive control (*GUS*-transformed rice seeds); (**d**) *GUS*-negative control (WT *P. volubilis* root tips); (**e**) root tips of regenerated *P. volubilis* after *Agrobacterium* inoculation; (**f**) bright-field image of *P. volubilis* chimeric plant with *DsRed2*-transformed roots; (**g**) bright-field image of WT *P. volubilis* plant; (**h**) fluorescence-field image of *P. volubilis* chimeric plant with *DsRed2*-transformed roots; (**i**) fluorescence-field image of WT *P. volubilis* plant; (**j**–**m**) frequency of positive plants, proportion of positive roots, number of positive roots, and rooting rate for different marker gene transformations in *P. volubilis*; the red arrows indicate the *GUS*-positive roots; the white arrows indicate the *DsRed2*-positive roots; bars = 1 cm; different lowercase letters indicate significant differences at 0.05 level (*p* < 0.05).

To evaluate the stability of the hairy root induction system in *P. volubilis*, two different reporting systems, *GUS* and *DsRed2*, were used for in planta *Agrobacterium* gel transformation. GUS staining was performed to assess the efficiency of *GUS*-positive root induction (Figure 3a,b). To improve the detection efficiency for positive plants, we excised the root tips (rather than the whole roots) of the plants to be tested for GUS staining using *GUS*-transformed rice seeds and WT *P. volubilis* root tips as the positive and negative controls, respectively (Figure 3c–e). Compared with GUS staining, DsRed2 inflorescence is a more convenient system for selecting positive hairy roots via a handheld fluorescence excitation light source (Figure 3f–i). Meanwhile, no significant differences were detected in the percentages of positive plants and the number and proportion of positive roots among the *GUS*, *DsRed2*, and *RUBY* gene transformation experiments (Figure 3j–m). These results suggest that the in planta *Agrobacterium* gel transformation system can efficiently and stably express various exogenous genes.

### 2.4. Applicability of the in Planta Agrobacterium Gel Transformation System in Different Explants of P. volubilis and Other Woody Plants

**Figure 4:**
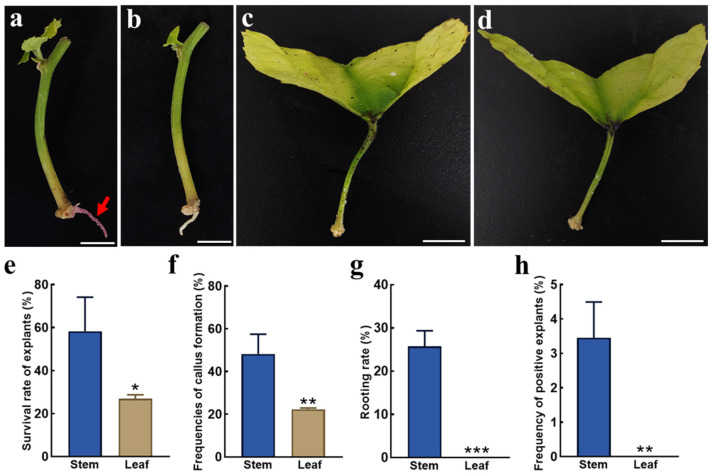
Applicability of the in planta *Agrobacterium* gel transformation system in different explants of plants. (**a**) *P. volubilis* stem with *RUBY*-transformed roots; (**b**) *P. volubilis* stem with WT roots; (**c**) *P. volubilis* leaf infected with *Agrobacterium*; (**d**) WT *P. volubilis* leaf; (**e**–**h**) the survival rate of explants, frequency of callus formation, rooting rate, and frequency of positive explants for stem segments and leaves; the red arrows indicate the *RUBY*-positive roots; bars = 0.5 cm; *, *p* < 0.05; **, *p* < 0.01; ***, *p* < 0.001.

To assess the applicability of the in planta *Agrobacterium* gel transformation system in various explants of *P. volubilis*, the stem segments and leaf petioles were also infected with the *Agrobacterium* gel (Figure 4a–d). At 30 days after inoculation, the survival rate, frequency of callus formation, and rooting rate of the stem segments were 58.29%, 48.23%, and 25.74%, respectively (Figure 4e–g). In contrast, the survival rate and callus formation rate of the leaves were 26.94% and 22.39%, respectively, with no root regeneration observed (Figure 4e–g). Ultimately, *RUBY*-transformed roots were successfully obtained in the stem segments, resulting in a positive explant frequency of 3.49% (Figure 4h). These results demonstrate that the hairy root induction system in *P. volubilis* is also applicable to stem segment-based transformation.

In addition, the in planta *Agrobacterium* gel transformation system successfully obtained *RUBY*-transformed roots in *P. corniculata*, *J. curcas*, *M. domestica*, and *M. alba* (Figure S3a–h). Among these, *P. corniculata* had the highest positive plant frequency (39.55%), while *Morus alba* had the lowest positive plant frequency (9.77%) (Figure S3i). However, most of the roots of *Morus alba* were positive, with a positive plant frequency of 75.56%, which was significantly greater than that of the other three plants (Figure S3j). Additionally, *P. corniculata* had the greatest number of positive roots and rooting rate (3.11% and 97.47%, respectively), whereas *Malus domestica* had the lowest number of positive roots and rooting rate (1.19% and 74.44%, respectively) (Figure S3k,i). These results indicate that the in planta *Agrobacterium* gel transformation system is suitable for hairy root induction in various woody plants.

### 2.5. Analysis of Disease Resistance Gene Function in Roots Using an in Planta Agrobacterium Gel Transformation System

**Figure 5:**
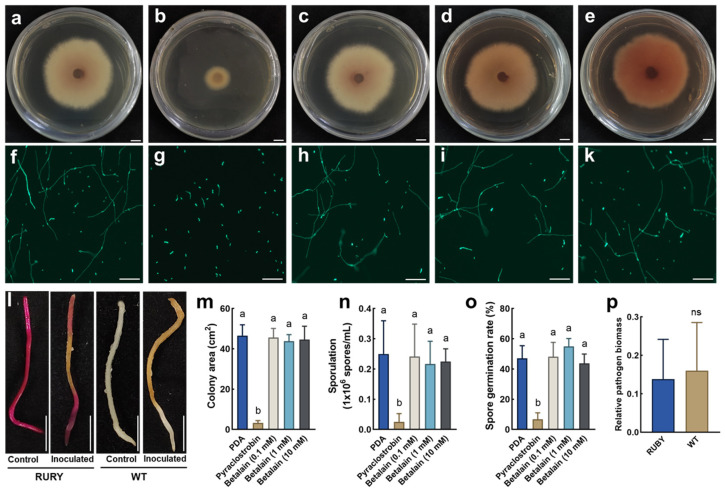
Effect of betalain on the growth, development, and pathogenicity of FoPvo1-GFP. (**a**–**e**) Growth of FoPvo1-GFP on PDA, pyraclostrobin (fungicide, 0.1 mM), and betalain (0.1, 1 and 10 mM) plates; bars = 1 cm; (**f**–**k**) germination of FoPvo1-GFP spores in sterile water, pyraclostrobin, and betalain (0.1, 1 and 10 mM) solutions; bars = 100 μm; (**l**) the phenotype of FoPvo1-GFP infection on the *RUBY* transgenic roots and WT roots; bars = 1 cm; (**m**–**o**) colony area, sporulation, and spore germination rate of FoPvo1-GFP after exogenous application of betalain; (**p**) relative pathogen biomass in the *RUBY* transgenic and WT roots; PDA, potato dextrose agar; FoPvo1-GFP, *Fusarium oxysporum* FoPvo1 overexpressing GFP; different lowercase letters indicate significant differences at 0.05 level (*p* < 0.05); ns, not significant.

The effects of betalain on the growth, development, and pathogenicity of a strain of *Fusarium oxysporum* FoPvo1 overexpressing GFP (FoPvo1-GFP) were evaluated by the treatment of FoPvo1-GFP with exogenous betalain in comparison with the fungicide or inoculation of the *RUBY* and WT roots with FoPvo1-GFP (Figure 5a–l). The results revealed that the fungicide pyraclostrobin inhibited FoPvo1-GFP growth, whereas no significant differences in the colony area, spore production, or spore germination rate were detected in response to betalain treatment (Figure 5m–o). Furthermore, the relative biomass of FoPvo1-GFP in the *RUBY* and WT roots after inoculation did not differ significantly (Figure 5p). These results indicate that betalain does not affect the growth, development, or pathogenicity of FoPvo1-GFP, suggesting that RUBY could be an ideal reporter system for the functional characterization of disease-related genes.

**Figure 6:**
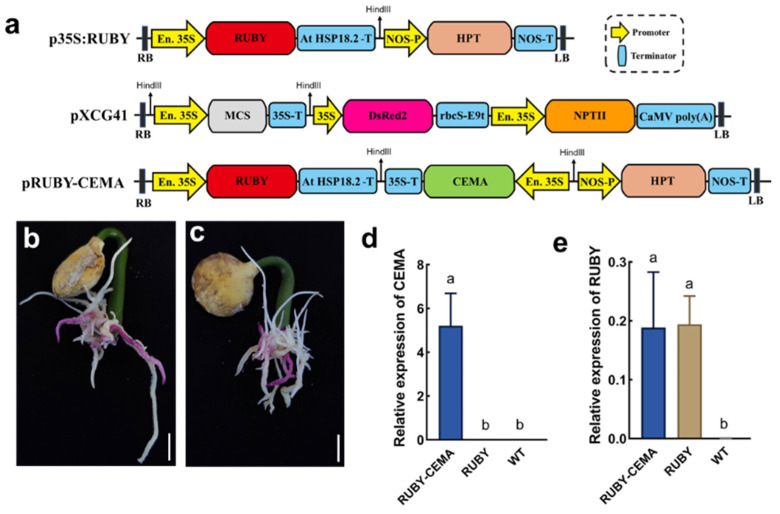
Simultaneous expression of *RUBY* and *CEMA* in *P. volubilis* Hairy Roots. (**a**) Schematic diagram of the pRUBY-CEMA-related vector (p35S:RUBY serves as the vector backbone, pXCG41 serves as the donor of the target gene expression cassette, and pRUBY-CEMA is the resulting derived vector); (**b**) *RUBY* + *CEMA* hairy roots; (**c**) *RUBY* hairy roots; (**d**) expression levels of the *CEMA* gene in the *RUBY* + *CEMA* hairy roots, *RUBY* hairy roots, and WT roots; (**e**) expression levels of the *RUBY* gene in the *RUBY* + *CEMA* hairy roots, *RUBY* hairy roots, and WT roots; bars = 1 cm; different lowercase letters indicate significant differences at 0.05 level (*p* < 0.05).

Next, we transformed pRUBY-CEMA (Figure 6a) into *P. volubilis* via the *Agrobacterium* gel transformation system to obtain transgenic hairy roots expressing both *RUBY* and the target gene *CEMA*. The results showed no significant morphological differences between the *RUBY* + *CEMA* and *RUBY* transgenic hairy roots (Figure 6b,c). A qRT-PCR analysis showed that both the *CEMA* and *RUBY* genes were highly expressed in the *RUBY* + *CEMA* hairy roots (Figure 6d,e), suggesting that pRUBY-CEMA can coexpress *RUBY* and *CEMA*.

**Figure 7:**
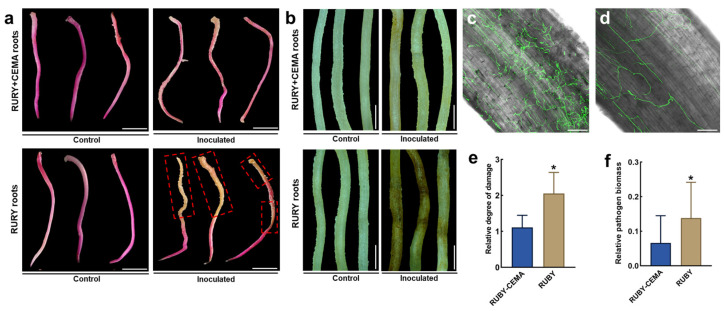
Overexpression of *CEMA* enhances *P. volubilis* hairy root resistance to FoPvo1-GFP. (**a**) the phenotype of FoPvo1-GFP infection in the *RUBY* + *CEMA* and *RUBY* hairy roots, with red boxes indicating bleaching areas; bars = 1 cm; (**b**) the phenotype of the *RUBY* + *CEMA* and *RUBY* hairy roots after decolorization; bars = 0.2 cm; (**c**) pathogen colonization in the *RUBY* hairy roots; bar = 200 μm; (**d**) pathogen colonization in the *RUBY* + *CEMA* hairy roots; bar = 200 μm; (**e**) damage level in the *RUBY* + *CEMA* and *RUBY* hairy roots; (**f**) relative pathogen biomass in the *RUBY* + *CEMA* and *RUBY* hairy roots; *, *p* < 0.05.

To further verify the function of *CEMA* in the regulation of disease resistance, FoPvo1-GFP was inoculated onto the *RUBY* + *CEMA* and *RUBY* hairy roots. The results showed that the red coloration in the mature and elongation zones of the *RUBY* hairy roots undergoes a significant fade following infection with FoPvo1-GFP. In contrast, the *RUBY* + *CEMA* hairy roots were resistant to FoPvo1-GFP, exhibiting much lower pathogen colonization (Figure 7a–d). Accordingly, the relative damage level and relative pathogen biomass in the *RUBY* + *CEMA* hairy roots were also significantly lower than those in the *RUBY* hairy roots (Figure 7e,f). Total extracts from the transgenic hairy roots were collected for the evaluation of CEMA’s antifungal activity, and the results showed that the colonies treated with the *RUBY* + *CEMA* hairy root extracts had significantly smaller diameters than those treated with the *RUBY* hairy root extracts (Figure S4). These results suggest that the overexpression of the antimicrobial peptide gene *CEMA* enhances the resistance of *P. volubilis* hairy roots to FoPvo1-GFP and that the use of in planta *Agrobacterium* gel transformation system to analyze disease resistance gene function in *P. volubilis* roots is feasible.

### 2.6. Gene Editing of P. volubilis Using the in Planta Agrobacterium Gel Transformation System

**Figure 8:**
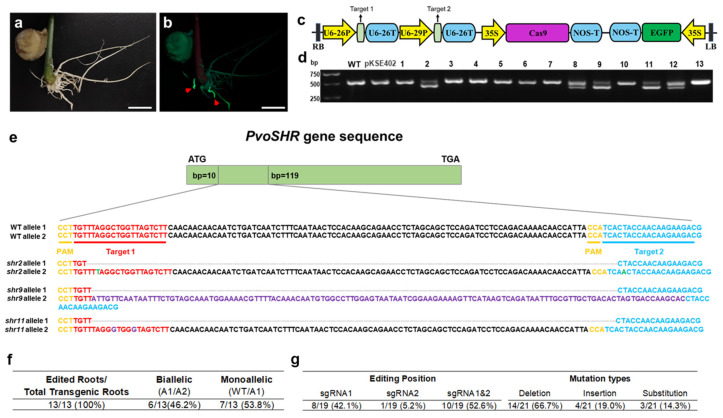
Gene editing of the *PvoSHR* gene in hairy roots using CRISPR-Cas9. (**a**,**b**) Chimeric plants of *P. volubilis* transformed with pKSE402-PvoSHR under bright field and green fluorescence fields (red arrows indicating the GFP-positive hairy roots); bars = 1 cm; (**c**) schematic diagram of the pKSE402-PvoSHR vector; (**d**) PCR gel electrophoresis of *PvoSHR* gene amplification (WT: WT roots; pKSE402: pKSE402 vector transformed hairy roots; 1–13: GFP-positive hairy roots transformed with pKSE402-PvoSHR); (**e**) three representative dual allele *PvoSHR* mutant sequences (yellow: PAM sequence; red: target 1 sequence; blue: target 2 sequence; green: base insertion; purple: base substitution; dashed line: base deletion); (**f**) efficiency of gene editing in hairy roots; (**g**) gene editing positions and types of mutation in hairy roots.

To evaluate the feasibility of the CRISPR-Cas9 system for gene editing in the roots of *P. volubilis*, we constructed a CRISPR-Cas9 vector (pKSE402-PvoSHR) targeting the *PvoSHR* gene and successfully obtained the hairy roots exhibiting GFP fluorescence via in planta *Agrobacterium*-mediated transformation method (Figure 8a–c). The PCR amplification of the *PvoSHR* gene from gDNA revealed that a few hairy root samples displayed double bands, indicating large deletions in one allele of the *PvoSHR* gene (Figure 8d). Further DNA sequencing analysis confirmed insertion, deletion, and substitution events between the two targeted sequences in the *PvoSHR* gene (Figure 8e).

Although editing events were detected in all the hairy roots, six roots exhibited biallelic gene editing, while the remaining seven roots displayed monoallelic editing, with no chimerism observed (Figure 8f, Table S2). A total of 21 editing events occurred in 13 roots, of which 42.1% occurred at sgRNA1, 5.2% at sgRNA2, and 56.2% occurred simultaneously at both sgRNA1 and sgRNA2. These editing events included gene deletions (66.7%), insertions (19.0%), and substitutions (14.3%). (Figure 8g, Table S2). Notably, when plant cells repair DNA breaks caused by sgRNA-Cas9 complex cleavage, substitution mutations due to mismatches typically involve only a few base pairs [25,26]. However, in this study, a 123-bp sequence replaced the original 110-bp sequence of the *shr9* allele 2. A homology comparison analysis suggested that this 123-bp sequence likely originated from the T-DNA region of the Ri plasmid pRi2659 from *A. rhizogenes* K599 (Figure S5).

**Figure 9:**
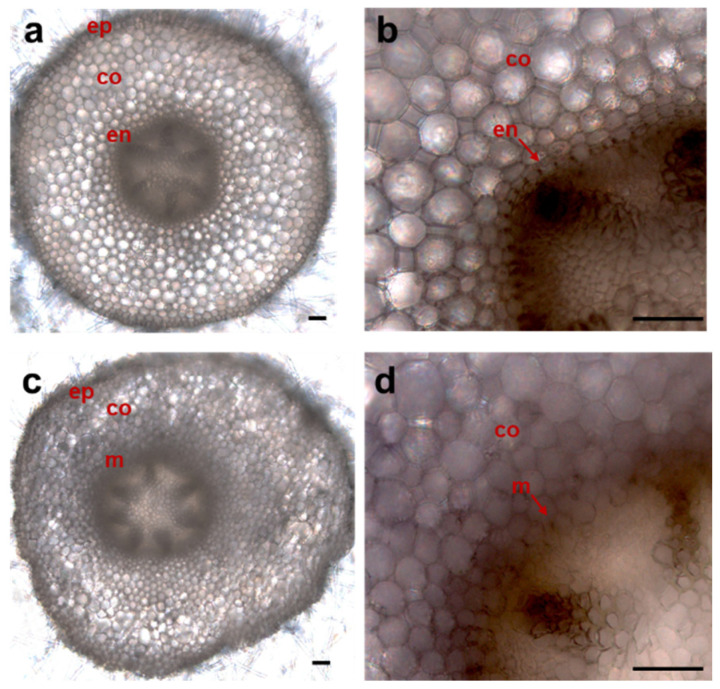
Cross-sections of *PvoSHR* gene-edited hairy roots. (**a**,**b**) Cross-section and local magnification of transgenic hairy roots expressing the empty vector pKSE402; (**c**,**d**) cross-section and local magnification of *shr* biallelic mutant hairy roots; ep, epidermis; co, cortex; en, endodermis; and m, mutant layer in *shr* roots; bars = 20 μm.

To determine whether the CRISPR-Cas9-mediated *PvoSHR* mutation affects the development of the endodermis in the roots of *P. volubilis*, we selected hairy roots with biallelic mutations for microscopic observation (Figure 9). The results indicated that the hairy roots expressing the empty vector (pKSE402) exhibited a distinct, single-layered endodermis (Figure 9a,b), whereas the *shr* biallelic mutant roots lacked a well-defined endodermis (Figure 9c,d). These findings suggest that the in planta *Agrobacterium* gel transformation system is efficient for gene function characterization by using CRISPR-Cas9.

## 3. Discussion

### 3.1. In Planta Agrobacterium-Mediated Transformation of P. volubilis Root System Provides Technical Support for Gene Function Research and Gene Editing

The large-scale implementation of telomere-to-telomere (T2T) genome sequencing has led to the discovery of numerous functional genes involved in regulating essential traits in forest trees [27]. However, due to the lack of an established genetic transformation protocol for woody plants, further analysis and application of important functional genes (such as the genes related to wood formation and stress resistance) remain challenging [28,29]. *A. rhizogenes*-mediated transformation, which results in the formation of hairy roots and chimeric plants, offers an ideal alternative for studying root biology and the interactions between roots and biotic/abiotic factors [10]. Numerous studies have explored hairy root induction systems for various tree species and fruit crops, including *Eucalyptus grandis*, *Vernicia fordii*, *Camellia sinensis* var. *sinensis*, *Prunus persica*, *Litchi chinensis*, *Salix purpurea*, *Malus domestica*, and *Actinidia chinensis*. The percentage of positive plants or explants, i.e., the percentage of plants or explants with transgenic hairy roots, reported in these studies ranged from 4% to 98.7% [17,30,31,32,33,34,35]. In our previous work, a tissue culture-based transformation method was developed for *P. volubilis*, achieving a percentage of positive plants of 6.42% [36]. However, this method requires a strict sterile environment and labor-intensive procedures. To address these challenges, Yu, et al. [37] explored an in planta transformation method to generate *RUBY*-positive hairy roots in *P. volubilis*; however, the induction efficiency is still relatively low. In this study, we systematically optimized the in planta *Agrobacterium*-mediated transformation system for *P. volubilis*, resulting in a significant increase in the percentage of positive plants (over 18.97%) (Figure 2), thus greatly facilitating the gene function studies in *P. volubilis*.

The hairy root transformation system enables the functional study of the genes involved in wood formation, secondary metabolite synthesis, and resistance to pests and diseases in forest trees [32,33,38,39,40,41,42]. In this study, we demonstrated that the hairy root induction system of *P. volubilis* can stably express multiple exogenous genes, including the reporter genes *RUBY*, *GUS*, and *DsRed2*, as well as the antimicrobial peptide-encoding gene *CEMA*. Additionally, we successfully integrated this system with CRISPR-Cas9 technology to knock out the *PvoSHR* gene in *P. volubilis*, resulting in a phenotype of epidermal layer deficiency (Figure 9). This finding suggests that similar to its homologs *PtaSHR* in *Populus tremula* × *alba* and *AtSHR* in *Arabidopsis thaliana*, the *PvoSHR* gene plays a conserved role in radial root differentiation and development [21,43]. The *Agrobacterium*-mediated transformation system established here provides a valuable tool for gene editing in *P. volubilis*.

A T-DNA sequence from the Ri plasmid pRi2659 of *A. rhizogenes* strain K599 was found to integrate into the double-strand DNA break site caused by CRISPR-Cas9 complex cutting, but the Ri T-DNA does not show any homology with the sequences flanking the DNA break (Figure S5), thereby excluding the possibility of a DNA insertion mediated by homologous recombination. It is known that during *Agrobacterium*-mediated plant transformation, T-DNA tends to integrate randomly into the plant genome, and its integration mechanism is not yet fully understood [44,45]. Previous studies have confirmed that T-DNA preferentially integrates into double-strand DNA breaks during the *A. tumefaciens* infection of plant cells [46,47]. Moreover, target sequence cleavage by the CRISPR-Cas9 complex can also lead to high-frequency T-DNA insertion [48]. Based on this principle, targeted T-DNA integration into the rice genome has been achieved via CRISPR-Cas9 [49]. This study also observed that the T-DNA sequence was integrated into the genome following double-strand DNA breaks induced by CRISPR-Cas9 in *P. volubilis*. However, in contrast to earlier studies demonstrating that T-DNA from *A. tumefaciens* Ti plasmids can integrate into CRISPR-Cas9 induced double-strand DNA breaks [48,49,50], this study is the first to report the knock-in of T-DNA from the *A. rhizogenes* Ri plasmid into such breaks (Figure S5). These results suggest that combining CRISPR-Cas9 with *A. rhizogenes* -mediated transformation enables targeted T-DNA knock-in in plants. Since each hairy root can generally be considered an independent transformation event [10], gene-edited hair roots can be efficiently obtained via *A. rhizogenes*-mediated transformation to evaluate the efficiency of targeted T-DNA integration and the development of targeted knock-in tools.

### 3.2. Gel Is an Ideal Carrier for A. rhizogenes and Helps Induce Hairy Roots

HPMC is a synthetic derivative of cellulose, modified by the addition of methyl and hydroxypropyl ether groups [51]. These modifications allow HPMC to dissolve in water at room temperature and quickly form a stable, highly transparent gel. As a result, HPMC is widely used as a thickening agent and emulsifier in the food, chemical, and pharmaceutical industries [52]. However, to date, no studies have reported the use of HPMC in *Agrobacterium* cultures to form a gel for infection and transformation. In this study, we innovatively combined *Agrobacterium* with HPMC to form an *Agrobacterium* gel for plant genetic transformation (Figure 1d). We compared the infection efficiency of three different forms of *Agrobacterium* (bacterial paste, suspension, and gel) (Figure S2) on *P. volubilis*, and found that the *Agrobacterium* gel infection method had the highest positive plant frequency without affecting the plant’s ability to regenerate roots (Figure 2). Unlike the *Agrobacterium* paste infection method, which requires several days of cultivation on agar plates to obtain sufficient *Agrobacterium* paste after activation [30,53], *Agrobacterium* gel can be rapidly prepared by adding HPMC to the activated *Agrobacterium* suspension for 10–20 min, significantly reducing the time required for hairy root induction. Moreover, as an ideal carrier for *Agrobacterium*, the gel can effectively encapsulate the wound and is not easily diluted by moisture in the potting substrate. By using the *Agrobacterium* gel infection method, we successfully obtained *RUBY*-positive adventitious roots in *P. volubilis*, *P. corniculata*, *J. curcas*, *M. domestica*, and *M. alba* (Figure 1 and Figure S3). Thus, this system enables fast, large-scale hairy root induction in *P. volubilis* and holds great potential for broad application.

### 3.3. Feasibility of RUBY as a Visual Screening Reporter for Transgenic Hairy Roots

To screen for transgenic hairy roots efficiently, this study compared the expression of three reporter genes (*RUBY*, *DsRed2*, and *GUS*) in *P. volubilis* hairy roots. The results showed that the *DsRed2* marker gene had the highest percentage of positive plants (22.71%), whereas the *RUBY* gene had the lowest percentage of positive plants (18.97%), although no significant difference was observed (Figure 3), which is consistent with the findings of Yang, et al. [54]. The relatively low positive plant frequency following *RUBY* gene transformation might be due to the occurrence of false negatives with the *RUBY* screening marker [37]. We also observed a similar phenomenon: uneven coloring in the root cap and mature zones of the same *RUBY* hairy root (Figure 1i) and the growth of white lateral roots on the red main root (Figure S6). Three enzymes in the *RUBY* system catalyze the synthesis of visually discernible red betalain using tyrosine as a substrate. Insufficient tyrosine supply, differences in *RUBY* gene expression levels, and the instability of betalain might contribute to false negatives in the *RUBY* screening system [37,55]. Therefore, the efficiency of hairy root induction may be underestimated when red *RUBY* roots are considered transgenic roots for statistical purposes. Compared with the WT plants, the plants expressing *RUBY* exhibited slight reductions in plant height and delayed flowering time [56], but no impact on disease resistance was observed. This study found that betalain had no significant effect on the growth, development, or pathogenicity of FoPvo1-GFP (Figure 5). When the antimicrobial peptide-encoding gene *CEMA* and *RUBY* were co-expressed in the same T-DNA region, the *CEMA* gene was stably expressed without affecting the *RUBY* expression levels (Figure 6). By screening for red roots, positive roots expressing the target gene can be efficiently obtained for disease-resistance gene function studies. Overall, *RUBY* is a convenient and efficient reporter for hairy root transformation.

## 4. Materials and Methods

### 4.1. Preparation of Plant Materials

The *Plukenetia volubilis* L. seeds were sourced from the planting base of the State Key Laboratory for Conservation and Utilization of Subtropical Agri-bioresources (Nanning, China). The dehulled seeds were soaked in sterile water for 48 h at 25 °C. The seed surface was subsequently sterilized with 75% (*v*/*v*) ethanol for 30 s, followed by immersion in a 10% (*v*/*v*) sodium hypochlorite solution for 20 min. The seeds were then washed three times with sterile distilled water and sown in sterile moistened perlite for germination at 28 °C. The seedlings that emerged after approximately 10 days of germination were used for hairy root induction experiments. Transgenic *Oryza sativa* expressing the reporter gene beta-glucuronidase (GUS) was purchased from Real-Times (Beijing) Biotechnology Co., Ltd. (RTU4042, Beijing, China).

### 4.2. Vector Construction

*CEMA* overexpression vector construction: The p35S:RUBY vector [57] was initially digested with *Hin*dIII (FD0504, Thermo Fisher Scientific, Waltham, MA, USA) to linearize the vector. The synthetic antimicrobial peptide-encoding gene *CEMA* [19] was then inserted into the multiple cloning site of the pXCG41 vector, which contains the vector backbone derived from pW501 [58] and the overexpression box derived from pOCA30 [59], creating the pXCG41-derived vector containing the *CEMA* sequence. Next, the pXCG41-derived vector was digested with *Hin*dIII to obtain the *CEMA* overexpression cassette, which was subsequently ligated with the linearized p35S:RUBY vector using T4 ligase (Thermo Fisher Scientific, Waltham, MA, USA) to generate the pRUBY-CEMA vector.

Construction of *PvoSHR*-Editing Vectors: The single guide RNA (sgRNA) was designed using the CRISPOR website (http://crispor.gi.ucsc.edu/crispor.py, accessed on 5 July 2024) (Concordet and Haeussler, 2018). Using the Golden Gate method [60], the pKSE402 vector [61,62] was ligated with two target sgRNAs (GAAGACTAACCAGCCTAAACA and GCGTCTTCTTGTTGGTAGTGA) derived from *PvoSHR* (GenBank accession no. PQ818118) to construct the pKSE402-PvoSHR knockout vector.

### 4.3. A. rhizogenes-Mediated Hairy Root Transformation System in P. volubilis

*Agrobacterium* gel infection method: A positive clone of *A. rhizogenes* K599 containing the p35S:RUBY vector (Addgene plasmid #160908, http://www.addgene.org/160908/, accessed on 20 March 2023) [57] was initially inoculated into 5 mL of yeast extract beef broth (YEB) medium supplemented with 50 mg·L^−1^ spectinomycin and 100 mg·L^−1^ streptomycin and cultured at 28 °C with shaking at 160 rpm for 48 h. After activation, 50 μL of the bacterial suspension was transferred to 50 mL of fresh YEB medium and incubated at 28 °C and 160 rpm until the optical density (OD) at 600 nm reached 0.6. The culture was then centrifuged (8000× *g* for 2 min), and the pellet was resuspended in 20 mL of buffer solution containing 200 μM acetosyringone (AS, CA1061, Coolaber, Beijing, China) and 1% sucrose. Next, 0.3–0.4 g of hydroxypropyl methylcellulose (HPMC) (H875054, Macklin, Shanghai, China) was added to achieve a final concentration of 1.5–2.0% (2% viscosity: 100,000 mPa·s), and the mixture was stirred for 10–20 min to form the *Agrobacterium* gel. The perlite (2–4 mm particle size) was placed in a 128-well tray and moistened with sterile water, and holes (approximately 1 cm in diameter and 2 cm in deep) were made in each well via a 1.5 mL centrifuge tube. The seedlings that had germinated for 10 days were selected and cut open at the plumule hook with a scalpel, and the basal hypocotyls and primary roots were then removed. The cut surface of the hypocotyls was immersed in the *A. rhizogenes* gel to completely cover the wound, and the seedlings were then inserted into the plug tray. The cells were covered with a clear plastic cover for moisture retention. The growth conditions were as follows: 16 h light/8 h dark photoperiod (30 μmol·m^−2^·s^−1^ light intensity), 25 °C and 95–100% humidity. The perlite needs to remain moist throughout *A. rhizogenes* co-cultivation. *RUBY* transgenic hairy roots were obtained after 20–30 days of induction.

*GUS*-transformed *P. volubilis* hairy roots were obtained using *Agrobacterium* containing the pCAMBIA2301-GUS [63]. The GUS staining solution was prepared following the manufacturer’s instructions for the GUS staining kit (RTU4032, Real-Times (Beijing) Biotechnology Co., Ltd., China) and aliquoted into 8-tube strips. One root tip of *P. volubilis* was placed into each tube, stained at 37 °C in the dark for 1 h, and then decolorized with 75% ethanol. The *GUS*-transformed rice grains were used as the positive control, whereas the WT *P. volubilis* roots served as the negative control. Since hairy roots generally originate from a single cell, each root is considered an independent transformation event [10]. *P. volubilis* hairy roots expressing the red fluorescent protein (DsRed2) gene were obtained via *Agrobacterium* containing the pW501 vector [58]. DsRed2 fluorescence signals were detected using a handheld fluorescence excitation light source (LUYOR-3415RG, LUYOR, Shanghai, China). The roots of WT *P. volubilis* were used as the negative control for DsRed2 fluorescence.

The efficiency of hairy root induction was assessed 30 days after treatment by calculating the positive plant frequency, positive root proportion, average number of positive roots, and rooting rate. The plants with positive hairy roots were considered positive for statistical analysis. The following formulas were used: frequency of positive plants (%) = (total number of positive plants/total number of plants) × 100; proportion of positive roots (%) = (total number of positive hairy roots/total number of roots on positive plants) × 100; number of positive roots per plant = total number of positive hairy roots/total number of positive plants; rooting rate (%) = (total number of rooting plants/total number of plants) × 100; survival rate of explants (%) = (total number of surviving explants/total number of explants) × 100; frequency of callus formation (%) = (total number of explants forming calli/total number of explants) × 100; and frequency of positive explants (%) = (total number of explants with positive roots/total number of explants) × 100. Each treatment was repeated three times, with a minimum of 20 explants per replicate.

*Agrobacterium* suspension infection method: After the *Agrobacterium* cells were resuspended, hydroxypropyl methylcellulose (HPMC) was not added. The cut surface of the lower hypocotyl was directly immersed in the bacterial suspension for infection. The subsequent steps were identical to those in the “*Agrobacterium* Gel Infection Method”.

*Agrobacterium* paste infection Method: A 2 mL aliquot of activated bacterial suspension (OD600 = 0.6) was centrifuged at 8000× *g* for 2 min. The supernatant (1.7 mL) was discarded, leaving 0.3 mL of the bacterial suspension. This suspension was thoroughly mixed and evenly spread onto YEB medium and then incubated at 28 °C for 24–48 h until the suspension thickened and formed a paste. The cut surface of the lower hypocotyl was then immersed in *Agrobacterium* paste for infection. The remaining procedures were the same as those in the “*Agrobacterium* Gel Infection Method”.

### 4.4. Molecular Identification and Expression Analysis of RUBY Hairy Roots

Genomic DNA (gDNA) was extracted from the hairy roots according to the kit manual provided with the HP Plant DNA Kit (D2485-00, Omega Bio-Tek, Norcross, GA, USA), followed by a PCR analysis for the molecular identification of the hairy roots. PCR amplification was conducted using the primers CYP76AD1-F1/R1, DODA-F1/R1, GT-F1/R1, and SmR-F1/R1 (Table S1). Primer design was performed using the online tool PrimerQuest (https://sg.idtdna.com/pages/tools/primerquest, accessed on 10 May 2023). The PCR conditions included initial denaturation at 94 °C for 5 min, followed by 30 amplification cycles: denaturation at 94 °C for 30 s, annealing at 55 °C for 30 s, and extension at 72 °C for 1 min. After the final amplification cycle, a 5 min extension was carried out at 72 °C. Quantitative real-time PCR was performed to analyze the expression levels of the *RUBY* expression cassette in both the WT and red hairy roots. Three hairy roots from the same plant were pooled and ground with liquid nitrogen. RNA was extracted using the EZNA Plant RNA Kit (R1027, Omega Bio-tek, Norcross, GA, USA), and complementary DNA (cDNA) was synthesized for use as a template in reverse transcription. The *P. volubilis* Actin gene (*PvoActin-7*) (GenBank accession no. PQ818117) was used as an internal reference [64] to normalize the cDNA quantity across samples. The reaction conditions were set according to the ChamQ Universal SYBR qPCR Master Mix (Q711, Vazyme, Nanjing, China) manual: pre-denaturation at 95 °C for 30 s, denaturation at 95 °C for 10 s, and annealing at 60 °C for 10 s, followed by 40 amplification cycles. The gene expression levels were calculated via the 2^−ΔΔCt^ method. All the reactions were performed in triplicate.

### 4.5. Determination of Betalain Content

The betalain content in the WT and red hairy roots was determined using the method described by Herbach, et al. [65]. In brief, the sample was thoroughly ground with distilled water to extract betalain and then centrifuged (8000× *g*, 10 min). The absorbance of the supernatant at 537 nm and 600 nm was then measured using the NanoDrop OneC Microvolume UV-Vis Spectrophotometer (Thermo Fisher Scientific, Waltham, MA, USA). The calculation method for betalain content is as follows: *c* = (Δ*A* × *F* × *M* × 1000 × *V*)/(*ε* × *l* × *m*). *c*—betalain content (mg·g^−1^); Δ*A*—difference in absorbance between 537 nm and 600 nm; *F*—dilution factor; *M*—molecular weight of betalain (550 g·mol^−1^); *V*—volume of the extract (L); *ε*—molar extinction coefficient of betalain (60,000 L·mol^−1^·cm^−1^); *l*—pathlength (1 cm); and *m*—mass of the sample to be tested (g). Three biological replicates were measured per sample.

### 4.6. Effects of Exogenous Additives on Hairy Root Efficiency in P. volubilis

Comparison of the effects of exogenous additives on hairy root induction efficiency: The effects of adding 200 μM acetosyringone (AS, CA1061, Coolaber, Beijing, China), 100 μM tenoxicam (TNX, T0909, Sigma, Ronkonkoma, NY, USA), and water (as a blank control) to *Agrobacterium* suspension buffer on hairy root induction efficiency in *P. volubilis* were analyzed using the method described in Section 4.3. The infection was performed via the *Agrobacterium* gel method, with perlite as the potting substrate.

### 4.7. Hairy Root Induction Methods for Stem Segments and Petioles of P. volubilis

Semilignified stem segments (1–2 months old) and mature leaves were harvested from one-year-old *P. volubilis* plants. *Agrobacterium* gel was applied to the cut surface of both the stem segments and petioles (with half of the leaf removed) to induce hairy root formation. The subsequent induction and cultivation procedures followed the method described in Section 4.3, “Induction of *P. volubilis RUBY* Transgenic Hairy Roots”.

### 4.8. Hairy Root Induction in P. corniculata, Malus Domestica, Morus Alba, and Jatropha Curcas

The methods for inducing hairy roots in *P. corniculata*, *Malus domestica*, *Morus alba*, and *Jatropha curcas* are similar to the *Agrobacterium* gel infection method described in Section 4.3, “Induction of Transgenic Hairy Roots of *P. volubilis RUBY*”. However, there are slight modifications in seedling age and explant treatment for the different species. For *P. corniculata*, the hairy root induction method used was identical to that used for *P. volubilis*. For *Malus domestica*, 7- to 10-day-old seedlings were selected, an incision was made at the junction of the root and the stem, the primary roots were discarded, and the subsequent treatment methods for *P. volubilis* were followed. For *Morus alba*, after a 128-well tray was filled with perlite, the perlite was moistened with a 5% sucrose solution, and the subsequent treatment methods for *Malus domestica* were followed. For *Jatropha curcas*, 5 to 7-day-old seedlings were cut at the hook of the plumule, the primary roots were discarded, and after the milky sap on the cut surface was absorbed with a tissue, the cut surface was covered with *Agrobacterium* gel, which induces hairy root formation. The subsequent treatment followed the methods used for *P. volubilis*.

### 4.9. Measurement of Colony Area, Spore Production, and Spore Germination Rate of F. oxysporum

A strain FoPvo1 of *F. oxysporum* was previously isolated from *P. volubilis* [66] and transformed its protoplasts with a GFP expression vector (pCT74) mediated by polyethylene glycol (PEG) [67], resulting in a strain with bright gfp fluorescence, which was designated as FoPvo1-GFP. Agar blocks (6 mm in diameter) containing FoPvo1-GFP were transferred to potato dextrose agar (PDA, negative control), PDA supplemented with 0.1 mM pyraclostrobin (PD20161392, BAINONG SIDA Bio-Tech, Weifang, China) (positive control), and PDA supplemented with 0.1 mM, 1 mM, or 10 mM betalain (R854445, Macklin, Shanghai, China). These plates were incubated in the dark at 28 °C for 5 days, and colony areas were measured using a cross method (length multiplied by width). Following incubation, 10 mL of sterile water was added to the plates to prepare the spore suspension. Spore counts were determined using a hemocytometer (Qiujing Biochemical Reagent Instrument Co., Ltd., Shanghai, China). The spore suspensions (5 × 10^5^ spores/mL) were treated with pyraclostrobin (0.1 mM) and betalain (0.1 mM, 1 mM, or 10 mM) and incubated in the dark at 28 °C for 12 h. A 10 μL aliquot of the spore suspension was dropped onto a hemocytometer, and more than 50 spores were counted per hemocytometer. The germination rate was calculated by dividing the number of germinated spores (with a germ tube length greater than half of the spore’s longest axis) by the total number of spores and then multiplying by 100.

### 4.10. Inoculation of P. volubilis Hairy Roots with FoPvo1-GFP

The FoPvo1-GFP spores were resuspended in sterile water to achieve a spore concentration of 2.5 × 10^6^ spores/mL. The roots of the *P. volubilis* plants were immersed in the spore suspension for 20 min, the seedlings were subsequently transplanted into culture cups (7 × 7 × 8 cm), and the roots were covered with sterile perlite. Following the addition of 30 mL of spore suspension around the root system of each seedling, the plants were co-cultivated for 48 h at 25 °C (95–100% humidity). Sterile water immersion was used as the control treatment.

After the hairy roots were co-cultivated with FoPvo1-GFP for 48 h, the root systems of the seedlings were thoroughly washed with sterile water. The hairy roots were then divided into two portions: one for calculating the relative root damage rate and observing the colonization of FoPvo1-GFP, and the other for determining the relative biomass of FoPvo1-GFP, with each treatment including at least 15 roots. Relative root damage rate: The iTOMEI method [68] was used to decolorize the roots, and the grayscale values of the control roots (inoculated with sterile water), inoculated roots (inoculated with the pathogen), and background were measured using the ImageJ software (ImageJ, 1.52e version). The relative root damage rate was calculated as the grayscale ratio: Relative root damage rate = (Grayscale value of inoculated root-Background grayscale value)/(Grayscale value of control root-Background grayscale value). Relative fungal biomass: gDNA was extracted from inoculated roots using the HP Plant DNA Kit (D2485-00, Omega Bio-Tek, Norcross, GA, USA). The *PvoActin-7* gene was used as the reference gene, and the *FoActin* gene (GenBank accession no. PQ878517) was used as the target gene. The relative expression of *FoActin* was measured using qPCR to assess the relative fungal biomass. The method for calculating relative expression was the same as that described in Section 4.4 “Molecular Identification and Expression Analysis of *RUBY* Hairy Roots”.

### 4.11. Observation of F. oxysporum Colonization by Laser Confocal Microscopy

The roots infected with FoPvo1-GFP, decolorized using the iTOMEI method [68], were placed on a glass slide and immersed in 30% (*v*/*v*) glycerol. A coverslip was gently placed to secure the sample. Colonization was observed immediately using a laser confocal microscope (Olympus Fluoview 3000, Olympus Corporation, Tokyo, Japan) with a UPLXAPO10X objective (10.0X, Numerical Aperture 0.4). GFP fluorescence was detected at 510 nm (High Sensitivity Spectral Detector 1 channel) with a 488 nm laser.

### 4.12. Antibacterial Activity Assay of Root Extracts on Agar Plates

Following the method described by Yevtushenko, Romero, Forward, Hancock, Kay and Misra [19], total extracts from *RUBY* and *RUBY* + *CEMA* transgenic hairy roots were collected and mixed with protease inhibitors (C510004, Sangon, Shanghai, China). Three 6 mm diameter holes were initially created in the PDA. Subsequently, 100 µL of unsolidified PDA was added to each hole to seal any potential gaps between the hole’s bottom and the Petri dish. Once the PDA solidified, the extracts were introduced into the respective holes to ensure simultaneous application without mixing. A 50 μL root extract or the fungicide pyraclostrobin (0.1 mM) (positive control) was mixed with 50 μL of *F. oxysporum* spore suspension (5 × 10^6^ spores/mL). The mixture was added to the holes, and the colony diameter was measured after incubating in the dark at 28 °C for 72 h. The experiment was repeated five times.

### 4.13. Statistical Analysis

Data analysis was performed using the SPSS statistical software (https://www.ibm.com/analytics/spss-statistics-software, accessed on 17 November 2024, IBM SPSS Statistics 24.0 version). One-way ANOVA was conducted for multiple comparisons between groups, followed by Tukey’s test (*p* < 0.05). Student’s t-test was used to analyze the differences between the experimental and control groups.

## 5. Conclusions

This study reports a highly efficient *A. rhizogenes*-mediated transformation system in the important oil crop *P. volubilis*. In combination with the *RUBY* visual reporter, an efficient and stable in planta *Agrobacterium* gel-mediated root transformation system for *P. volubilis* was established. This system can be used for the functional studies of the genes related to disease resistance and root development in *P. volubilis*, and provides an essential reference for gene function studies in other tree species that lack established genetic transformation systems.

## Data Availability

Data are contained within the article and Supplementary Materials.

## Supplementary Materials

The following supporting information can be downloaded at https://www.mdpi.com/article/10.3390/ijms26062496/s1.
