# Supplementary material for: Agrobacterium rhizogenes-Mediated Hairy Root Genetic Transformation Using Agrobacterium Gel Inoculation and RUBY Reporter Enables Efficient Gene Function Analysis in Sacha Inchi (Plukenetia volubilis)
Source: Int J Mol Sci. 2025 Mar 11;26(6):2496. doi: 10.3390/ijms26062496 (PMC11941831; doi:10.3390/ijms26062496)
Supplement: Supplementary file 1 [file ijms-26-02496-s001.zip › ijms-3491046-supplementary/Figure S1-S6, and Table S1_Final Proofreading_LK_Xu.docx]

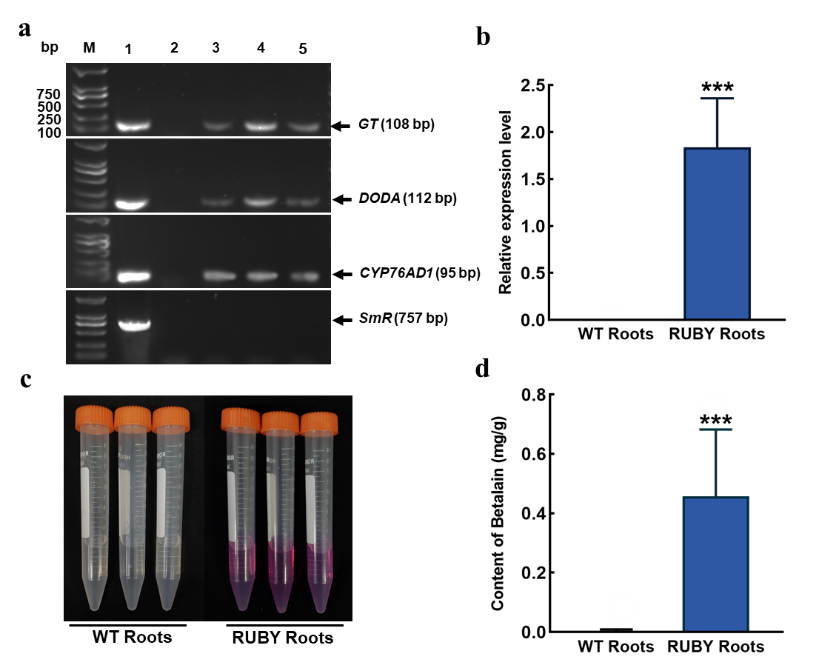


**Figure S1.** Identification of *RUBY* transgenic hairy roots of *P. volubilis*. (**a**) PCR electrophoresis profile (M: Marker; Lane 1: Positive control, p35S:RUBY vector; Lane 2: Negative control, WT root; Lanes 3-5: Red hairy roots); (**b**) The expression level of *RUBY* gene in WT and red hairy roots; (**c**) Water extract from WT and *RUBY* hairy roots; (**d**) Betalain content in WT and *RUBY* hairy roots; *GT*, *glucosyltransferase*; *DODA*, *_L-_3,4-dihydroxyphenylalanine 4,5-dioxygenase*; *CYP76AD1*, a P450 oxygenase gene; *SmR*, streptomycin-resistance gene; ***, *p* < 0.001.


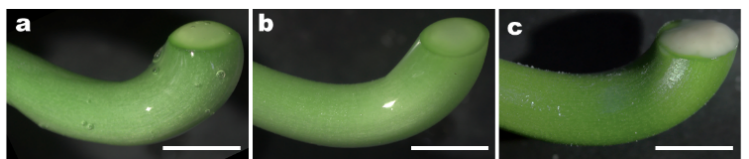


**Figure S2.** Three methods of *Agrobacterium* infection on *P. volubilis* hypocotyls. (**a**) *Agrobacterium* gel; (**b**) *Agrobacterium* suspension; (**c**) *Agrobacterium* paste; bars = 1 cm.


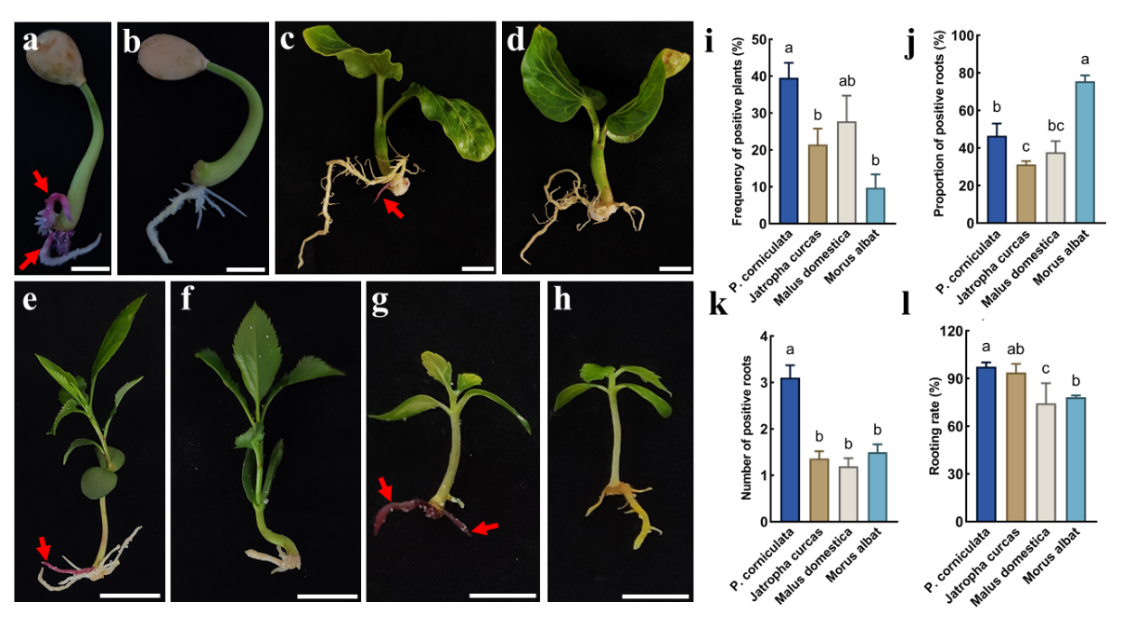


**Figure S3.** Applicability of *Agrobacterium* gel + *RUBY* root induction system in different woody plants. (**a, b**) *RUBY*-transgenic and WT roots in *P. corniculata*; (**c, d**) *RUBY*-transgenic and WT roots in *Jatropha curcas*; (**e, f**) *RUBY*-transgenic and WT roots in *Malus domestica*; (**g, h**) *RUBY*-transgenic and WT roots in *Morus alba*; (**i-l**) Frequency of positive plants, proportion of positive roots, number of positive roots, and rooting rate in four woody plants; red arrows indicate *RUBY*-positive roots; bars = 1 cm.


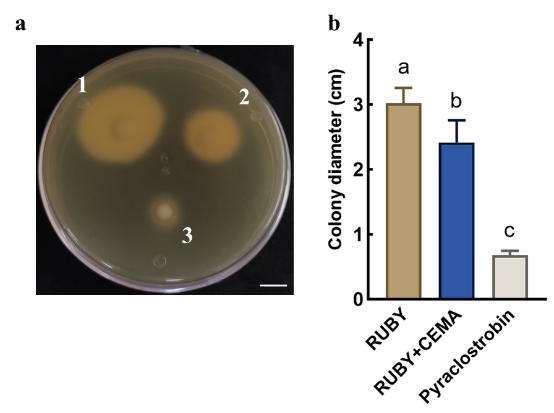


**Figure S4.** Effects of *RUBY* + *CEMA* and *RUBY* root extracts on FoPvo1-GFP growth. (**a**) The growth of FoPvo1-GFP on plates containing root extract and fungicide (1: *RUBY* root extract; 2: *RUBY* + *CEMA* root extract; 3: pyraclostrobin); (**b**) FoPvo1-GFP colony diameter; bar = 1 cm; different lowercase letters indicate significant differences at 0.05 level (*p* < 0.05).


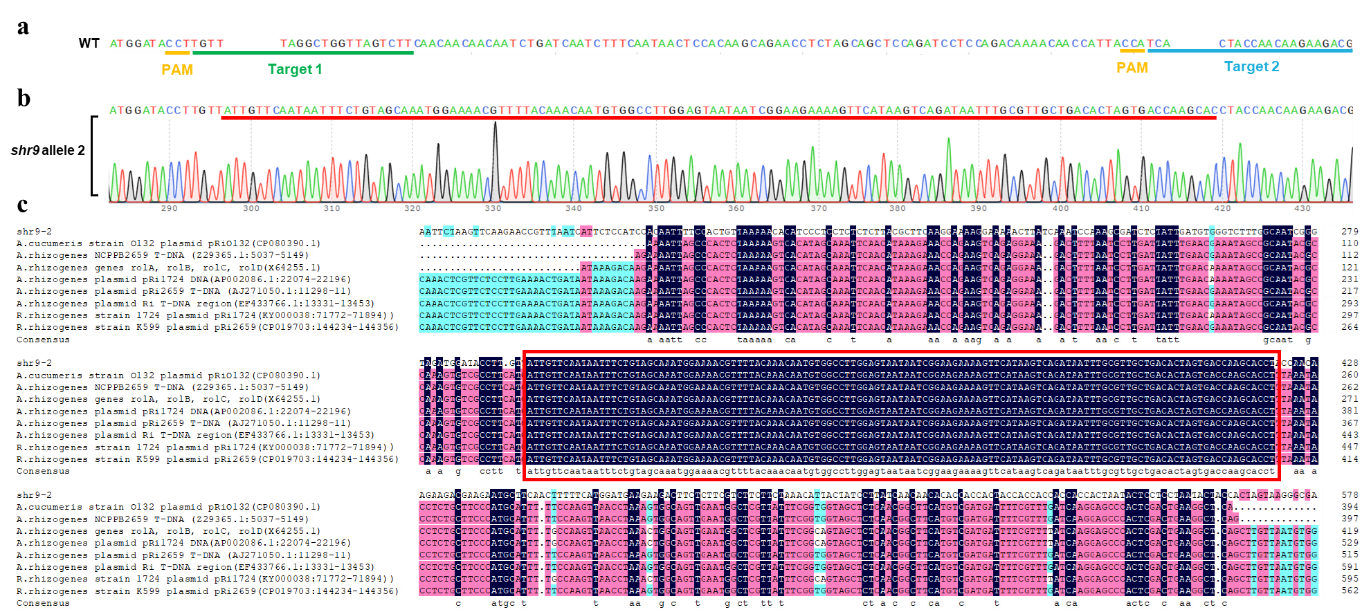


**Figure S5.** Integration of *Agrobacterium* T-DNA sequence into the *P. volubilis* genome. (**a**) Target sites of *PvoSHR* gene and PAM sequences; (**b**) *shr9* allele 2 sequence; the red underline indicates the large fragment sequence inserted into *shr9* allele 2; (**c**) Alignment of large fragment sequence inserted into *shr9* allele 2 (shr9-2) with T-DNA sequences of *Agrobacterium* Ri plasmids. Black background indicates 100% homology, pink background indicates >75% homology but <100%, blue background indicates >50% homology but <75%, and the red box indicates a region with identical sequence.


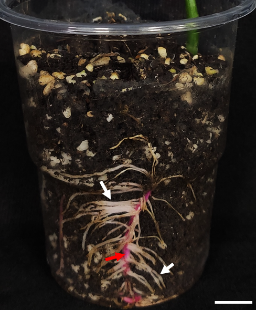


**Figure S6.** Accumulation of betalain in the primary and lateral roots of *RUBY* transgenic plants is uneven. Red arrows indicate *RUBY*-positive primary roots; white arrows indicate white lateral roots growing from *RUBY*-positive primary roots; bar = 1 cm.

**Table S1** PCR primers used in this study

| Primer name | GenBank ID of  target gene | Sequence (5'→3') | Length (bp) | Purpose |
| --- | --- | --- | --- | --- |
| CYP76AD1-F1 | HQ656023.1 | CGATACCACATCCTCCACATTC | 95 | *RUBY*-positive root identification |
| CYP76AD1-R1 |  | GGACCTGCTTGATTTCCTCTT |  |  |
| DODA-F1 | HQ656027.1 | TTGCGAGAAAGGTGGAAGAG | 112 | *RUBY*-positive root identification |
| DODA-R1 |  | GGATACATGAGCATGAGTGGAA |  |  |
| GT-F1 | AB182643.1 | GGCATCGAGAACACCGATAAG | 108 | *RUBY*-positive root identification |
| GT-R1 |  | GCTGAAATGCCTGGAGATGTA |  |  |
| SmR-F1 | KF687969.1 | CGAAGTATCGACTCAACTATC | 757 | *RUBY*-positive root identification |
| SmR-R1 |  | CTTGGTGATCTCGCCTTTCA |  |  |
| PvoActin-7-F1 | PQ818117 | CCAGAAGTCTTGTTCCAGCCATCTC | 185 | Relative biomass detection |
| PvoActin-7-R1 |  | GCGGTGATCTCCTTGCTCATACG |  |  |
| FoActin-F1 | PQ878517 | CCGAGGCTCCCATCAACC | 194 | Relative biomass detection |
| FoActin-R1 |  | GGCGAAACCCTCGTAAATGG |  |  |
| PvoSHR-DT1-BsF1 | PQ818118 | ATATATGGTCTCGATTGAAGACTAACCAGCCTAAACAGTT | 626 | Construction of pKSE402-PvoSHR |
| PvoSHR-DT2-BsR1 |  | ATTATTGGTCTCGAAACTCACTACCAACAAGAAGACGCAA |  |  |
| PvoSHR-DT1-F1 | PQ818118 | TGAAGACTAACCAGCCTAAACAGTTTTAGAGCTAGAAATAGC | 597 | Construction of pKSE402-PvoSHR |
| PvoSHR-DT2-R1 |  | AACTCACTACCAACAAGAAGACGCAATCTCTTAGTCGACTCTAC |  |  |
| PvoSHR-cxF1 | PQ818118 | TGAAGCGAGGATTCCACCTT | 581 | Amplification of *PvoSHR* |
| PvoSHR-cxR1 |  | ACTAGTGGTAGTATTAGGAGG |  |  |

**Table S2.** Details of mutations detected in *shr* roots (Supplied as a separate document).
